# 73-year-old Female with Syncope and Motor Vehicle Collision

**DOI:** 10.5811/cpcem.2021.7.53384

**Published:** 2021-11-01

**Authors:** Kevin Flanagan, Zachary D.W. Dezman, Karl J. Dachroeden, Laura J. Bontempo

**Affiliations:** * University of Maryland School of Medicine, Department of Emergency Medicine, Baltimore, Maryland; † University of Maryland, Department of Epidemiology and Public Health, Baltimore, Maryland; ‡ University of Maryland Medical Center, Department of Emergency Medicine, Baltimore, Maryland

**Keywords:** syncope, takotsubo, CPC

## Abstract

**Introduction:**

Patients with traumatic injuries can be difficult to assess, and their evaluation often evolves in the emergency department (ED). We describe how an ED attending physician member developed a differential diagnosis for this presentation, arrived at a suspected diagnosis, and what test he proposed to prove his hypothesis.

**Case Presentation:**

This clinicopathological case presentation details the initial assessment and management of a 73-year-old female who presented to the ED following a motor vehicle collision precipitated by a syncopal episode.

**Conclusion:**

The final surprising diagnosis is then revealed.

## CASE PRESENTATION (DR. DACHROEDEN)

A 73-year-old female was brought by emergency medical services (EMS) to the emergency department (ED) after a motor vehicle collision (MVC). She was driving when her car crashed into a streetlamp at 40 miles per hour. The EMS team reported that the pole intruded into the passenger compartment and the patient required a prolonged extrication. She had a Glasgow Coma Scale score (GCS) of 11 when EMS arrived at the scene, which improved to a 15 after repeated evaluations. She was conscious, alert, and oriented at the scene and during transport.

The patient had difficulty recalling the events before the collision. She remembered feeling “like I was having a panic attack” just before the crash, and she did not recall the car impacting the streetlamp. In the ED, she complained of substernal chest pressure. The patient’s history was limited due to her continued difficulty recalling the events of the accident. The patient’s son was contacted and he stated that she lives alone and is active and independent. He stated that she had experienced syncopal episodes over the last several years, all associated with paroxysms of panic when driving, and she had undergone extensive workups without any clear etiology identified. She had magnetic resonance imaging (MRI) of her brain with and without contrast four years prior for stroke-like symptoms after a syncopal event. Per his report, no abnormalities were found.

The patient had a past medical history of anxiety, migraines, Hashimoto thyroiditis, and hyperlipidemia. She had been diagnosed with breast cancer in 2012, which was treated with a left mastectomy followed by five years of tamoxifen and letrozole treatment. She had a family history of dementia, diabetes, and heart disease. She denied using tobacco, alcohol, or illicit substances. She was prescribed, and was taking, the following medications: levothyroxine 25 micrograms daily; atorvastatin 20 milligrams (mg) nightly; rizatriptan 10 mg as needed for headaches; and multivitamins.

On physical exam, the patient’s temperature was 36.5° Celsius, her heart rate was 92 beats per minute, and her blood pressure was 142/72 millimeters of mercury. She was breathing at a rate of 21 breaths per minute with an oxygen saturation of 98% on room air. She weighed 125 pounds and was 5 feet, 3 inches tall, with a body mass index of 22.1 kilograms per meter squared. She did not appear to be in any acute distress, was healthy appearing, and was oriented to her name, date, and location. Her head was normocephalic and atraumatic; she had no Battle sign, raccoon eyes, or other signs of trauma. She had hearing aids in place bilaterally. She had normal extraocular movements with equal pupils that were reactive to light. She had a cervical collar in place. Later, when it was safe to remove her collar, her neck had a normal range of motion without rigidity or pain.

On auscultation, her heart had a regular S1 and S2 at a normal rate and was without murmurs. She had normal pulses bilaterally. Her lungs were clear without wheezing or rhonchi, and she was noted to have normal effort without respiratory distress. Her abdomen was soft and flat, without tenderness or guarding. Her musculoskeletal exam showed an overall normal range of motion without deformities. She had no step-offs of the spinous processes or tenderness along the midline spine. The remainder of her skeletal exam only revealed tenderness over the left wrist. She had a capillary refill of less than two seconds. On her skin exam, she had minor contusions to the left upper chest near the axilla with superficial abrasions along the left wrist. She was not diaphoretic. Neurologically she had no cranial nerve deficits, with no motor weakness or sensory deficits peripherally. She had a normal gait and normal coordination as well. She could not recall her birth date and had slow verbalization with some word-finding difficulty. Psychiatrically, she was normal in mood and behavior.

**Image 1:**
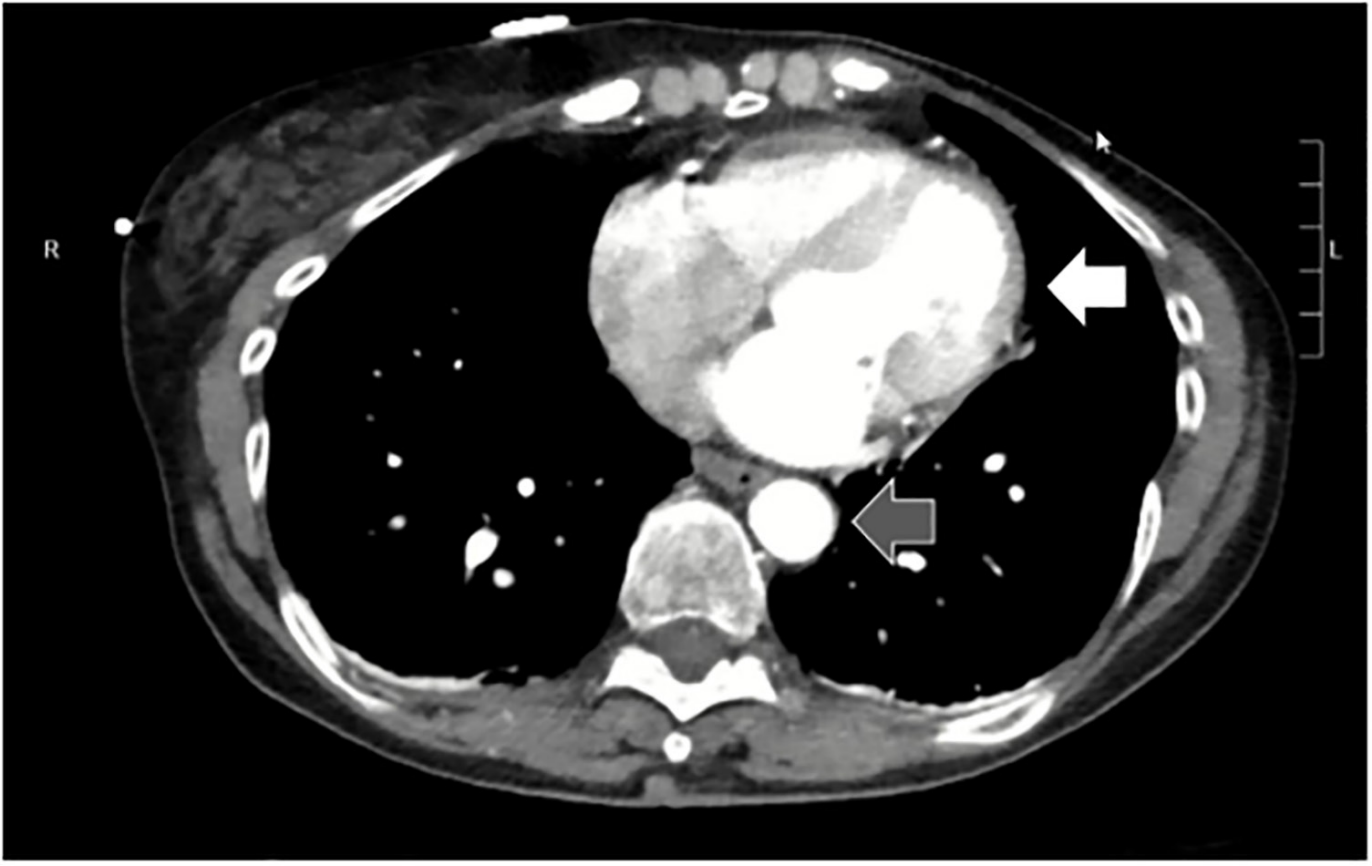
Transverse view of heart (white arrow) and descending aorta (gray arrow) taken from the computed tomography angiogram of the chest of a 73-year-old female with syncope and subsequent motor vehicle collision.

Point-of care-ultrasound did not find evidence of free fluid within the abdominal or thoracic cavities and was negative for a pericardial effusion. Point-of-care ultrasound examination of her chest revealed normal lung sliding bilaterally. Chest radiograph showed no acute abnormalities. A radiograph of her pelvis showed no fracture or malalignment of the bones. A computed tomography (CT) of her head and brain showed no intracranial injury. A CT of the cervical spine and chest with contrast in the arterial phase was performed, which showed normal vertebral body height and alignment without fracture. The CT did show some mild atherosclerosis of the carotid and vertebral arteries, but no vascular injury and no obvious narrowing. Her trachea and mainstem bronchi were patent, she had no mediastinal abnormalities, the thoracic aorta was normal in appearance, and she had no pericardial effusion or pneumothorax (Image 1).

**Image 2:**
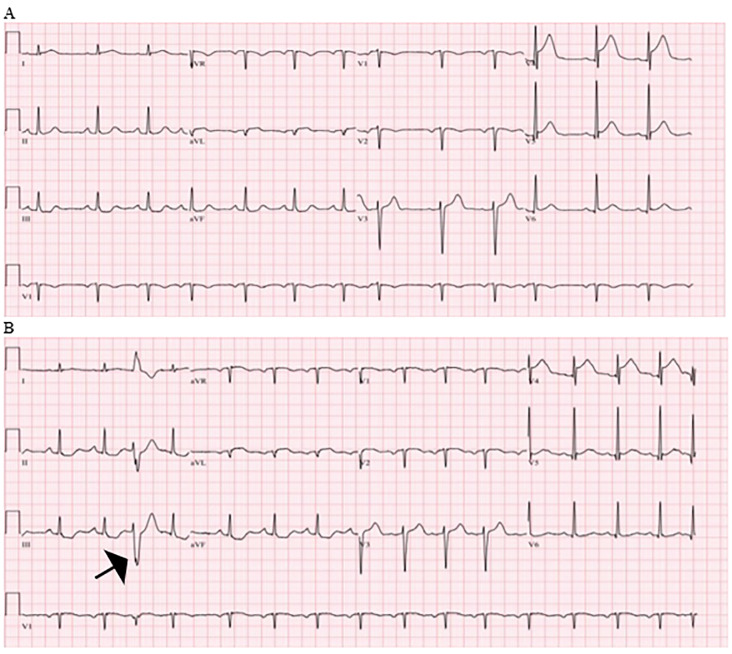
Initial (A) and repeat (B) electrocardiogram of a 73-year-old female with syncope and subsequent motor vehicle collision. Noted is a premature ventricular complex (arrow) and ST-segment depression in the inferior leads

A CT of her abdomen and pelvis with contrast in the venous phase was also performed, which showed patent vasculature including a normal caliber aorta and inferior vena cava, normal appearing spleen, pancreas, adrenal glands and kidneys/ureters, a small hiatal hernia but otherwise normal appearing bowel, and no ascites or pneumoperitoneum. The radiologist did not find any fractures on her imaging or any soft tissue injuries. Initial laboratory studies (Table 1), laboratory studies after administration of intravenous fluids (Table 2), and the patient’s electrocardiograms (ECG) (Images 2) are shown. A test was then performed and a diagnosis was made.

## CASE DISCUSSION (DR. FLANAGAN)

Patients with traumatic injuries can be difficult to assess and their evaluation often evolves in the emergency department (ED). This case highlights how a meaningful history of present illness can be a priceless asset in the evaluation of a complicated trauma patient.

This case focuses on a 73-year-old female, restrained driver with confusion after being in a MVC. She later develops respiratory symptoms and chest pain while in the ED. While the history-taking is limited by her ongoing confusion, she describes a preceding syncopal event with a prodrome of anxiety.

This patient must be first evaluated through the lens of acute injury; so I applied the principles of Advanced Trauma Life Support. Her initial assessment was reassuring because there were no injuries identified that require immediate intervention. I calculated a GCS of 15 from the physical exam provided. Her vital signs were within normal limits and she is in no acute distress. This completes the primary trauma survey (airway, breathing, circulation and disability), but the mechanism of injury and several features of this case worry me.

My primary concern is her cardiopulmonary symptoms, which could be caused by an occult diaphragmatic, aortic, myocardial, or pulmonary injury, even though her vital signs are currently normal. Secondly, her disorientation, dysphasia, and lethargy are alarming. Despite a reassuring GCS, these findings could be a sign of an intracranial hemorrhage or due to an injury elsewhere causing poor cerebral perfusion.

This patient clearly requires a rapid and thorough evaluation. The case presenter states the patient underwent an extended focused abdominal sonography in trauma (eFAST) examination, which did not show any free fluid. But the diagnostic capability of the eFAST exam is limited in hemodynamically stable patients;, therefore, this patient needed to undergo CT and computed tomography angiography (CTA) imaging.^1^ No traumatic injuries were identified on imaging, and her laboratory evaluation shows no evidence of anemia, severe metabolic derangement, or end organ damage.

The combination of physical examination and advanced imaging studies excludes acute traumatic injuries from my diagnosis. Her preceding symptoms of anxiety and the resulting loss of consciousness now begs attention. Her son also noted multiple previous similar episodes of syncope, all while driving, with similar prodromal symptoms described as a “panic attack.” This sudden and transient loss of consciousness brings into question several etiologies including new-onset epilepsy and vascular and cardiogenic causes of syncope.

Her loss of consciousness could represent a seizure. Her symptoms of confusion, lethargy, and word-finding difficulty may represent a postictal state. Lactate can be elevated briefly after a seizure. Her prodrome of anxiety could represent a seizure with aura. But neither the patient nor her son reported a history of tonic-clonic like activity, and the patient did not experience any loss of bowel or bladder continence. Her brain imaging did not identify any significant ischemia, masses, or other parenchymal abnormalities, which are the most common causes of new-onset epilepsy in the elderly. Seizure, therefore, is lower on my differential diagnosis.

Vascular causes of syncope include aortic or carotid dissection, vertebrobasilar insufficiency, pulmonary embolism, and posterior strokes. However, none of these causes fit clinically with the presentation or the patient’s recurrent prodrome. Furthermore, the patient’s physical examination, radiology studies and prior brain MRI would have identified these conditions.

Cardiogenic causes of syncope are best summarized by three possibilities: arrythmia, structural heart disease, and ischemia. These are all best initially evaluated via electrocardiogram (ECG). Looking at the patient’s first ECG, I do not see any of the following findings suggesting an arrythmia:

- Arrhythmogenic intervals, such as a prolonged QT or high-degree atrioventricular blocks;
- Waveform morphologies concerning for pre-excitation pathways, Brugada syndrome, or arrhythmogenic right ventricular dysplasia.

There are no features on the ECG concerning for common structural heart diseases such as right or left ventricular hypertrophy. There is non-specific ST-segment depression inferiorly in leads II, III and aVF along with concave J-point elevations in V4 and V5. Her repeat ECG has similar features, but the ST-segment depression along the inferior leads is more pronounced and there are some dynamic changes along the precordium (lower amplitude T-waves V5 and V6), which is concerning for ischemia (Image 2).

I examined the patient’s lab work for clues as to arrhythmogenic causes of syncope. There is no evidence of abnormalities of her serum potassium, calcium, or magnesium, which would place the patient at risk for an arrythmia. Her troponins were not elevated on her initial lab results, making cardiac ischemia a less likely cause, but given the short interval since symptoms onset, these tests will need to be repeated.

While the patient’s CTA imaging is limited because it captures a single moment in time, findings on CTA imaging are consistent with an underlying cardiac cause of the patient’s presentation. The inferior vena cava, right heart, and pulmonary vascular circuit all appear prominent in the imaging and suggest some degree of left heart dysfunction. There are no overt masses that would represent a dynamic obstruction such as a left atrial myxoma, and I see no thickening of the left ventricular myocardium as would be found in patients with diastolic dysfunction. The shape of the left ventricle does appear abnormal, however. Normally, the left ventricle is conical in shape and tapers in diameter along the long axis. The luminal contour of the left ventricle in our patient’s CTA appears dilated along the apical portion with no identifiable obstruction along the outflow tract.

In summary, this is a 73-year-old female with cardiopulmonary complaints following a MVC that was preceded by an anxiety-provoked syncopal event. Her evaluation shows no traumatic injuries but demonstrates dynamic ECG changes and abnormal cardiovascular findings on CTA imaging. Piecing together the clinical features of this presentation with her completed evaluation, I am concerned that there is an underlying cardiogenic etiology of her symptoms. Laboratory evaluation shows no current evidence of myocardial damage. The CTA imaging infers an underlying cardiac dysfunction with obvious prominence of the pulmonary and right-sided vasculature. Together with the abnormal shape of the left ventricle, these findings suggest a non-ischemic cardiomyopathy. The recurrence of these syncopal events, each time with prodromal anxiety is particularly interesting and suggests that her anxiety may be a causal factor of her symptoms.

When considered alongside her cardiopulmonary symptoms, her dynamic ECG changes, and the anatomical abnormalities identified along the apical portion of her left ventricle; I conclude that her diagnosis is takotsubo cardiomyopathy. This diagnosis would be confirmed by an echocardiogram.

## CASE OUTCOME (DR. DACHROEDEN)

**Image 3:**
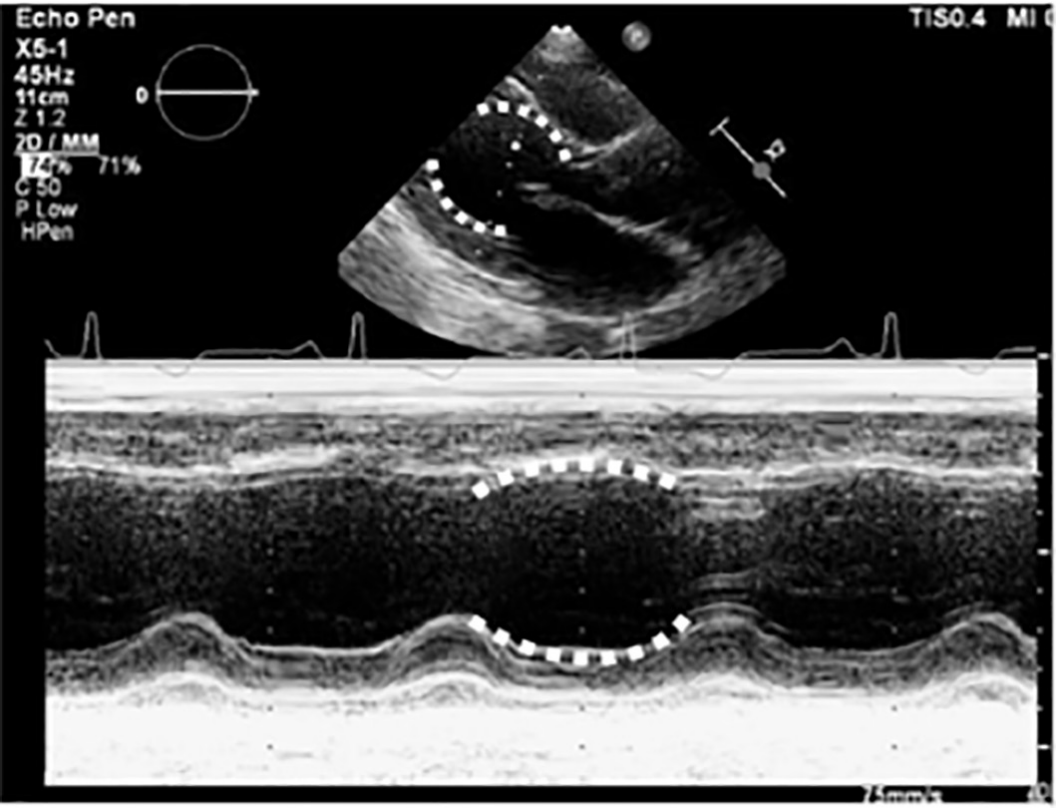
Transthoracic echocardiogram of 73-year-old female at the left sternal border showing apical ballooning (white dotted line) in the long axis (top) and m-mode (bottom).

The study that led to the diagnosis was an echocardiogram (Image 3), which showed an ejection fraction (EF) of 40% with apical ballooning. These findings, plus the lack of any significant coronary artery disease found on emergent percutaneous coronary angiography, led to a diagnosis of takotsubo cardiomyopathy. In the ED, the patient received 500 milliliters of normal saline for her lactic acidosis and she was started on aspirin, a beta-blocker, and an angiotensin-converting enzyme inhibitor. On her second hospital day, a cardiac MRI confirmed the diagnosis. She was seen by psychiatry who started her on buspirone, and after one week of hospitalization she was discharged. At her five-month follow-up visit she had complete return of her cardiac function.

## RESIDENT DISCUSSION (DR. DACHROEDEN)

Takotsubo cardiomyopathy, or apical ballooning syndrome (ABS), is characterized by regional systolic dysfunction of the left ventricle (LV). It was first described in Japan in 1990.^2^,^3^ The true incidence is still unknown, although it does appear to have greater incidence in women, the elderly, and those with thyroid disorders.^4^ It is estimated to occur in 1–2% of cases of suspected acute coronary syndrome (ACS) or ST-elevation myocardial infarction (STEMI),^5^,^6^ as well as in a significant percentage of critically ill patients.^7^

The most accepted pathophysiology of ABS is an excess of catecholamines leading to coronary artery spasm and myocardial stunning.^8^ While an acute emotional stress is the classic trigger associated with ABS, causing it to also be referred to as “broken heart syndrome,” it is most commonly triggered by a severe medical illness.^4^,^9^ The Mayo Clinic has proposed four criteria, all of which must be present to make the diagnosis of ABS: 1) transient left ventricular systolic dysfunction that involves more than the distribution of a single coronary artery; 2) absence of obstructive coronary disease/angiographic evidence of plaque rupture OR vessel disease that is not in the distribution of the wall motion abnormalities; 3) new ECG changes or modest elevation in troponin; and 4) absence of pheochromocytoma or myocarditis.^12^ Note that the diagnosis can be made without a trigger being identified.

Cardiac dysfunction is usually documented using a combination of echocardiography, angiography, and cardiac MRI. A STEMI is present 43% of the time and is typically located in the anterior leads.^4^,^11^ Cardiac biomarker testing is typically elevated to a significant degree in these patients, including troponin and brain natriuretic peptide levels.^4^ The degree of ECG change or troponin elevation unfortunately has not been sufficient to differentiate between ACS and ABS but, interestingly, the brain natriuretic peptide levels do appear to exceed the levels found in their ACS-matched counterparts.

Treatment of this disease is primarily focused on supportive therapy with conservative management; most cases resolve in one to four weeks.^13^ The most common complications are the development of cardiogenic shock, heart failure and thromboembolism. Interestingly, the development of shock does not appear to be related to a patient’s level of systolic dysfunction^4^,^14^ but may be related to the presence of LV outflow tract (LVOT) obstruction.^15^ An LVOT obstruction is important to identify to optimize management, as it changes the expected treatment. Again, given the relatively recent discovery of this disease there are few trials comparing treatment in LVOT obstruction, however, based on knowledge of hypertrophic cardiomyopathy treatment, it does appear that a similar approach should be pursued. This includes using beta-blockers to improve filling and decrease obstruction, the avoidance of preload reduction^16^ and balloon pump therapy in severe shock states that are refractory to initial management.

No formal studies have been performed to identify ideal heart failure management strategies in ABS. It is therefore recommended that patients be managed by standard protocols including diuresis^17^ except when LVOT obstruction is present, as previously discussed. Given that the pathophysiology of this disease is suspected to be catecholamine related, inotropic agents that act through sympathetic mechanisms are thought to potentially worsen the disease, although they are recommended for temporization. Finally, there is a risk of intraventricular thrombus formation that may embolize; therefore, despite limited data, there are recommendations for anticoagulant therapy. If a thrombus is seen, then anticoagulation should continue for three months or until cardiac function returns and the thrombus resolves. If a thrombus is not seen, then the recommendation is anticoagulation for three months or until the significant cardiac dysfunction improves, whichever is shortest.

While ABS is primarily thought of as a transient disease, there are associated risks of in-hospital complications and morbidity after discharge, both of which more commonly occur in males. When compared to their ACS-matched counterparts, ABS patients had similar or increased rates of serious in-hospital complication such as cardiogenic shock, need for vasopressors, ventilation (invasive and noninvasive), and cardiopulmonary resuscitation.^4^ These events do appear to be more common in younger patients and those with a physical trigger, as well as those with underlying psychiatric disease, or a baseline EF of under 45%.^4^ There is a 7.1% risk of major adverse cardiac or cerebrovascular events such as death, stroke, or transient ischemic attack in the first 30 days after admission, and again men are more affected than women.^4^ Long-term follow-up of these patients demonstrates an all-cause mortality of 5.6% per patient-year and a rate of major adverse cardiac and cerebrovascular events of 9.9% per patient-year and, once again, men have worse outcomes than women.^4^ Unfortunately, beta-blocker therapy has not been shown to improve survival rates at one year, although angiotensin-converting enzyme inhibitors were associated with improved survival.^4^

## FINAL DIAGNOSIS

Takotsubo cardiomyopathy (apical ballooning syndrome).

## KEY TEACHING POINTS

Takotsubo cardiomyopathy is also referred to as apical ballooning syndrome (ABS) and, more colloquially, as broken heart syndrome.

In ABS, an excess of catecholamines is thought to lead to coronary artery spasm and myocardial stunning, which can present as syncope and/or a STEMI.

A severe medical illness or emotional stress can trigger ABS.

If ABS is suspected, look for left ventricular outflow tract obstruction on echocardiogram, as this finding alters management.

In the absence of left ventricular outflow obstruction, treatment is supportive and the workup follows normal care patterns for suspected acute coronary syndrome.

## Figures and Tables

**Table 1:** Initial laboratory results of 73-year-old female with syncope and subsequent motor vehicle collision.

Test Name	Result	Reference Range
Complete blood cell count		
White blood cells	10.2 K/mcL	4.5 – 11 K/mcL
Hemoglobin	13.2 g/dL	11.9 – 15.7 g/dL
Hematocrit	41.2%	35.0 – 45.0%
Platelets	200 K/mcL	153 – 367 K/mcL
Complete metabolic panel		
Sodium	131 mmol/L	136 – 145 mmol/L
Potassium	hemolyzed	3.5 – 5.1 mmol/L
Chloride	97 mmol/L	98 – 107 mmol/L
Carbon dioxide	18 mmol/L	21 –30 mmol/L
Blood urea nitrogen	14 mg/dL	7 – 17 mg/dL
Creatinine	0.7 mg/dL	0.52 – 1.04 mg/dL
Glucose	195 mg/dL	70 – 99 mg/dL
Calcium	10.3 mg/dL	8.6 – 10.2 mg/dL
Magnesium	2.4 mg/dL	1.6 – 2.6 mg/dL
Phosphorus	4.0 mg/dL	2.5 – 4.5 mg/dL
Total protein	7.4 g/dL	6.3 – 8.2 g/dL
Albumin	4.3 g/dL	3.2 – 4.6 g/dL
Aspartate aminotransferase	46 units/L	14 – 36 units/L
Alanine aminotransferase	24 units/L	0 – 34 units/L
Total bilirubin	0.8 mg/dL	0.3 – 1.2 mg/dL
Alkaline phosphatase	73 units/L	38 – 126 units/L
Additional labs		
Lactate	8.6 mmol/L	0.5 – 1.6 mmol/L
Troponin	0.02 ng/mL	<=0.06 ng/mL
Partial thromboplastin time	21 seconds	25 – 38 seconds
Prothrombin time	12.3 seconds	12.1–15.0 seconds
International normalized ratio	0.9	unknown
Fibrinogen	354mg/dL	216 – 438 mg/dL
Thyroid stimulating hormone	1.08 mlU/L	0.47–4.68 mlU/L
Urinalysis		
Color	Yellow	
Appearance	Clear	
Specific gravity	>1.040	1.002 – 1.030
pH	5.0	5.0 – 8.0
Glucose	Negative	Negative
Bilirubin	Negative	Negative
Urobilinogen	Negative	Negative
Ketones	Trace	Negative
Blood	Negative	Negative
Protein	Negative	Negative
Nitrite	Negative	Negative
Leukocyte esterase	1+	Negative
White blood cells	6 – 10 / hpf	0 – 5 /hpf
Red blood cells	6 – 10 /hpf	0 – 2/hpf
Squamous epithelial cells	Negative	Negative
Bacteria	Negative	Negative

*K*, thousand; *mcL*, microliter; *g*, gram; *dL*, deciliter; *mmol*, millimoles; *L*, liter; *mg*, milligram; *ng*, nanogram; *mIU*, milli-international units; *hpf*, high powered field.

**Table 2:** Repeat laboratory results taken two hours after arrival of a 73-year-old female with syncope and subsequent motor vehicle collision.

Test Name	Result	Reference range
Complete metabolic panel		
Sodium	132 mmol/L	136 – 145 mmol/L
Potassium	3.4 mmol/L	3.5 – 5.1 mmol/L
Chloride	97 mmol/L	98 – 107 mmol/L
Carbon dioxide	23 mmol/L	21 –30 mmol/L
Blood urea nitrogen	15 mg/dL	7 – 17 mg/dL
Creatinine	0.7 mg/dL	0.52 – 1.04 mg/dL
Glucose	183 mg/dL	70 – 99 mg/dL
Calcium	9.3 mg/dL	8.6 – 10.2 mg/dL
Total protein	5.9 g/dL	6.3 – 8.2 g/dL
Albumin	3.3 g/dL	3.2 – 4.6 g/dL
Aspartate aminotransferase	32 units/L	14 – 36 units/L
Alanine aminotransferase	20 units/L	0 – 34 units/L
Total bilirubin	0.4 mg/dL	0.3 – 1.2 mg/dL
Alkaline phosphatase	62 units/L	38 – 126 units/L
Additional labs		
Lactate	3.6 mmol/L	0.5 – 1.6 mmol/L
Partial thromboplastin time	25 seconds	25 – 38 seconds
Prothrombin time	13.5 seconds	12.1–15.0 seconds
International normalized ratio	1.0	0.9 – 1.1
Fibrinogen	296 mg/dL	216 – 438 mg/dL

*Mmol*, millimoles; *L*, liter; *mg*, milligram; *dL*, deciliter; *g*, gram.
